# Supplementary material for: Plasma-Etched Vertically Aligned CNTs with Enhanced Antibacterial Power
Source: Nanomaterials (Basel). 2023 Mar 16;13(6):1081. doi: 10.3390/nano13061081 (PMC10054568; doi:10.3390/nano13061081)
Supplement: Supplementary file 1 [file nanomaterials-13-01081-s001.zip › nanomaterials-2225029-supplementary.pdf]

Supplementary Materials

Plasma-Etched Vertically Aligned CNTs with Enhanced Antibacterial Power

Emily Schifano [1,2], Gianluca Cavoto [3,4], Francesco Pandolfi [4], Giorgio Pettinari [5], Alice Apponi [6], Alessandro Ruocco [6], Daniela Uccelletti [1,2], Ilaria Rago [3,4,*]

[1] Dipartimento di Biologia e Biotecnologia “C. Darwin”, Sapienza University of Rome, Piazzale Aldo Moro 5, 00185 Rome, Italy
[2] SNN Lab, Sapienza Nanotechnology & Nano-Science Laboratory, Sapienza University of Rome, 00100 Rome, Italy
[3] Dipartimento di Fisica, Sapienza Università di Roma, Piazzale Aldo Moro 2, 00185 Rome, Italy
[4] INFN Sezione di Roma, Piazzale Aldo Moro 2, 00185 Rome, Italy
[5] Istituto di Fotonica e Nanotecnologie, CNR-IFN, Via del Fosso del Cavaliere 100, 00133 Rome, Italy
[6] Dipartimento di Scienze, Università Degli Studi Roma Tre and INFN Sezione di Roma Tre, Via della Vasca Navale 84, 00146 Rome, Italy
* Correspondence: ilaria.rago@uniroma1.it

Table S1. Optimization of plasma treatment conditions.

Identifier	Ar [sccm]	O_2 [sccm]	Pressure [mTorr]	Power [W]	Time [min]
1	0	195	1	33	1
2	0	66	40	100	1
3	0	66	40	100	2
4	52	14	40	80	1
5	52	14	40	80	5
6	20	5	70	50	5
7	20	5	100	50	5
8	10	2	200	50	5
9	10	2	200	35	5

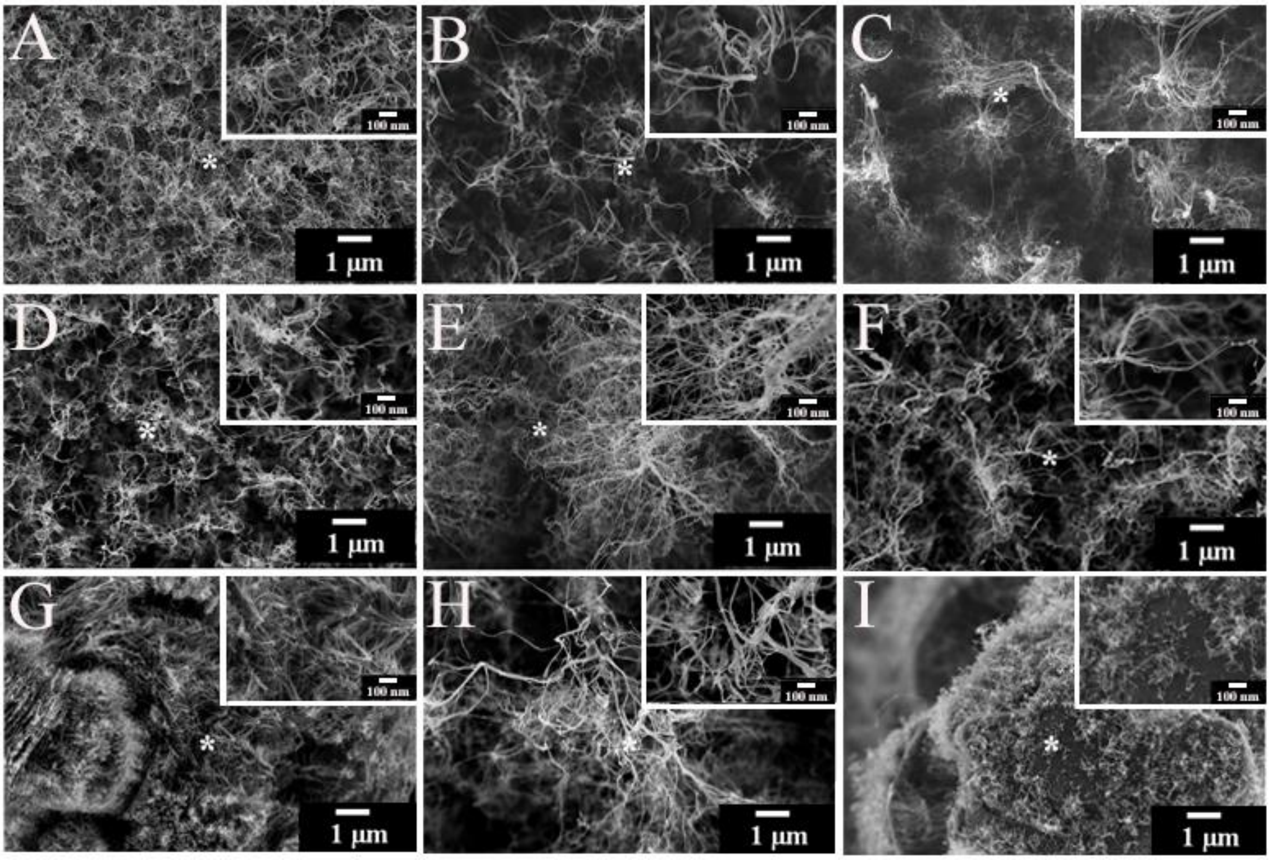

Figure S1. (A-I) Top view SEM micrographs of VA-CNTs modified with different plasma etching conditions showing the morphology of: (A) sample 1 in Table S1 where no significant morphological alterations occur and the crust layer seems to be still present over the surface of CNT mats (*i.e.* "light" etching), (B-C) samples identified as 2 and 3 in Table S1 in which the uniform layer of randomly oriented CNTs (*i.e.* crust layer) switches to a more spiky CNTs arrangement, categorized as "aggressive" etching, (D-H) samples 4-8, upon which significant morphological and structural alterations emerge, (I) sample 9 where the crust is removed and the structural integrity of CNT forests is maintained, identified as "modest" etching.
